# Electrochemical and Spectroscopic Studies of Zinc Oxide in an Eco-Friendly Deep Eutectic Solvent for Zn Electrodeposition

**DOI:** 10.1149/1945-7111/adda79

**Published:** 2025-05-28

**Authors:** Kazem Mohammadzadeh, Uttam Kumer Roy, Abhishek Lahiri

**Affiliations:** 1 Department of Chemical Engineering, College of Engineering, Design and Physical Science, Brunel University of London, Uxbridge, Middlesex UB8 3PH, United Kingdom; 2 Water Engineering and Development Centre, School of Architecture, Building and Civil Engineering, Loughborough University, Loughborough, Leicestershire LE11 3TU, United Kingdom

## Abstract

Zinc oxide (ZnO) is a waste produced from end-of-life Zinc-air batteries. Recycling of these spent batteries is important to reduce the pressure on primary zinc sources and to mitigate environmental pollution. To address the recyclability of waste ZnO from different sources, here we have studied ZnO dissolution and Zn electrodeposition in deep eutectic solvents (DES) of betaine hydrochloride (Betaine-HCl) and formic acid (FA) using spectroscopic and electrochemical techniques. Additionally, the effect of water in the DES on the dissolution of ZnO and its electrochemical and spectroscopic behaviour was also studied. Solubility of ZnO in DES was found to be 95 g l^−1^ which decreased slightly to 85 g l^−1^ in the DES containing 10% water. Fourier transform infrared and Raman studies revealed that ZnO dissolved in DES by forming a [ZnCl_3_FA]^−^ complex. Electrochemical studies showed that Zn deposit morphology and structure varied with water content in the DES. A uniform Zn deposit was achieved in ZnO-DES mixture whereas the presence of water gave a porous morphology. Thus, this study revealed an easy and eco-friendly route to recycle ZnO.

Deep eutectic solvents (DESs) are promising alternative to organic solvents and conventional ionic liquids (ILs) due to its low cost and low toxicity.^1,2^ DESs are typically formed of hydrogen bond acceptors (HBAs) and hydrogen bond donors (HBDs), which interacts through hydrogen-bond networks, resulting in a drastic reduction in the melting point of the mixture in comparison to its individual components.^1^ DESs offer excellent properties such as low vapour pressure, high thermal stability, relatively large electrochemical window and significant solubility for metal salts.^2–6^ Due to their tuneable nature and ease of preparation, DESs have emerged in various applications such as biocatalysis, metal processing, and electrochemistry.^1,2^

Zinc is a widely used non-ferrous metal. Due to its excellent corrosion resistance, good compressive ductility, and fine wear resistance, it is a prime candidate for sustainable energy storage system and galvanisation.^6^ Traditional aqueous-based electrolytes for zinc deposition suffer from various challenges such as hydrogen evolution reactions (HER), passivation, and dendrite formation, resulting in low coulombic efficiency and compromised deposit morphology.^3,7,8^ These limitations necessitate the development of alternative electrolytes for efficient zinc deposition and recovery.

DESs offer a promising solution for the challenges posed by aqueous electrolytes for recycling ZnO and zinc electrodeposition. Recent studies have demonstrated the efficiency of DESs in achieving dendrite-free zinc deposition.^8^ Dissolution of various metal oxides have been investigated in DES. For example, Abbott et al.^9^ investigated the solubility of transition metal oxides including ZnO in three DESs electrolytes based on malonic acid (MA), urea (U) and ethylene glycol (EG) with choline chloride (ChCl) at a ratio of 1:1, 1:2 and 2:1, respectively, and reported low solubility of ZnO (0.46 mg ml^−1^) in ethylene glycol-based DES (ChCl: EG) and the high solubility (16.2 mg ml^−1^) in malonic acid-based DES (ChCl: MA), which is due to the predominate formation of chlorometalate species (ZnCl_3_^−^). Rodriguez et al.,^10^ showed that the solubility of ZnO could be increased by changing the HBDs in DES and achieved a maximum solubility of ZnO (57.7 mg ml^−1^) in p-toluenesulfonic acid-based DES (Ptsa-ChCl) (2:1). Using a combination of EG and MA as HBD’s, Jangir et al.^11^ showed the possibility of improving the dissolution of ZnO to 94.25 mg ml^−1^ at 323.15 K. Similar to Abbott et al., Zhu et al.^12^ reported the solubility of ZnO to be ~24.3 mg ml^−1^ in ChCl-urea/EG based DES from which Zn was electrodeposited. On changing the HBA to 1-allyl-3-methylimidazolium chloride ([Amim]Cl) with urea as the HBD at 1:1 ratio, Zheng et al.^13^ showed an improvement in the dissolution of ZnO to ~83 mg ml^−1^ at 373 K from which Zn was electrodeposited. Similarly, He et al. and Liu et al.,^14,15^ confirmed that Zn electro-deposition can be achieved at temperatures of 353 and 373 K on using 2:1 urea/1-ethyl-3-methyllimidazolium chloride to dissolve the ZnO and electrodeposit Zn.

Compared to imidazolium HBA, DES consisting of betaine hydrochloride, urea and the glycerol at a ratio of 1:4:2.5 was used to dissolve 30 different metal oxides and subsequently electrodeposit metals from the corresponding DES solutions.^16^ It was shown that the solubility of ZnO in DES was ~42 mg ml^−1^ at 423 K and Zn could be electro-deposited at 333 K. However, the study revealed that DES or leached ZnO in DES solution were unstable at room temperature. Therefore, developing appropriate DESs with improved stability is important which can be used several times in order to recycle the metal oxides.

Here, we have studied betaine hydrochloride-formic acid-based DES to dissolve ZnO and electrochemically deposited Zn from the electrolyte. As DES are hydrophilic in nature, we have also investigated the effect of water concentration in DES on the dissolution of ZnO, and its influence on Zn electrodeposition. The dissolution mechanism and Zn speciation were studied using FTIR and Raman spectroscopy. The electrochemical behaviour of Zn in the presence of water in DES were investigated using cyclic voltammetry (CV). SEM and XRD were employed to identify the electrodeposited Zn and to understand their elemental compositions and surface morphologies.

## Material and Methods

Zinc Oxide (99.9%) and formic acid were purchased from Thermo Fisher Scientific. Betaine Hydrochloride (>99%) was bought from Sigma-Aldrich. All chemicals’ reagents were used without further purification.

The DESs were prepared following earlier method described by Luo et al.,^17^ with some modification. In brief, Betaine hydrochloride (Betaine-HCl) and formic acid (FA) at a molar ratio of 1:6 was used for the synthesis of DES with the addition of 10%, 20%, 30%, 40% w/w of deionised water (W). The mixtures were stirred using a magnetic stirrer in an oil bath at 333 K until a homogeneous colourless liquid was formed. To the synthesised DES, different amounts of ZnO were gradually added and the mixtures were stirred continuously with a magnetic stirrer at 333 K up to 4 h. The stability of dissolved ZnO in DESs at room temperature was investigated for 10 days. The viscosity of DESs was measured at 294 K by automatic rolling-ball micro-viscometer (Anton Paar GmbH, Graz, Austria). Each measurement was performed in triplicate, and the average value was reported. Fourier-transform infrared (FTIR) spectrometer (IRSpirit-X, Japan) was used to analyse the liquid samples. The Raman measurements were conducted using a confocal Raman microscope which was equipped with a 514 nm laser (steller-REN) and using a diffraction grating of 1800 lines/mm with a Renishaw CCD camera as the detector.

The CV and electrodeposition experiments were carried out in a three-electrode system with graphite sheet (RS pro) used as working and counter electrodes, and a platinum wire used as the quasi-reference electrode. The electrochemical experiments were performed using a potentiostat (Biologic VSP-3e) that was controlled by a desktop computer with a Biologic software (EC lab). The cyclic voltammetry (CV) of DES and solutions of ZnO in DES were measured between −1.6 to +0.8 V with a scan rate of 10 mV s^−1^ at room temperature. Constant potential electrodeposition was conducted in DES solution at room temperature at different voltages.

The crystal structure of the electrodeposited zinc was analysed by X-ray diffractometer (Bruker D8 Advance XRD, Germany) using a monochromatic Cu kα radiation at a wavelength of λ = 1.5406 and a LynxEye™ silicon strip detector. The surface morphology of the zinc coating was examined by scanning electron microscope (Jeol IT200LV, USA) which is coupled with an energy dispersive spectroscopy (EDS) (Oxford EDS) as the detector.

## Results and Discussion

### Characterisation of DES based electrolyte

The viscosity of various DES-water mixtures at room temperature (294 K) is shown in Table I from which it is evident that viscosity of the DES decreases with increase in water content. The DES without water was not measured as DES was found to be unstable (see Fig. S1) at room temperature (294 K) after a day. The reduction in the viscosity of DES with the addition of water can be attributed to the change in the hydrogen bond networking between HBD and HBA.^1,11,18,19^

The solubility of ZnO in DES-water mixtures at 333 K indicates that DESs can dissolve relatively high concentrations of ZnO (Table I). The dissolution of ZnO in the DES without water at 333 K was found to be 95 g l^−1^. Interestingly, it was observed that although the DES was unstable at room temperature, on addition of ZnO in the DES, the electrolyte became stable at room temperature. Additionally, it was observed that the presence of small concentration of H_2_O molecules (10%) in the DES did not change the solubility of ZnO (85 g l^−1^) by a significant amount which could be attributed to embedding H_2_O molecules through strong interactions (van der Waals, H-bonds, electrostatic,)^19,20^ inside and at the periphery of the DES without forming any separate H_2_O clusters. On further increasing the water concentration (20%–40%) a decrease in the solubility of ZnO (Table I) is observed which can be attributed to the dissociation of DES into individual ions or ion-pairs.^19^ On comparing our results with ChCl-based DES systems reported by other authors, it is evident that higher concentration of ZnO can be dissolved in Betaine-HCl-based DES. Compared to results obtained here, we found that similar dissolution concentration was achieved by Zheng et al. in imidazolium-based DES.^13^ However, high concentration of ZnO dissolution was shown by Liu et al.,^21^ who reported that ~2.5 mole l^−1^ ZnO could be dissolved in protic ionic liquid of 1-methylimidazolium trifluoromethylsulfonate ([MIm]TfO) at 394 K.

However, both imidazolium based aprotic and protic electrolytes are more expensive compared to DES electrolytes and therefore the suitability of the electrolyte for recycling purpose will be difficult.

### ZnO dissolution mechanism

**Figure 1:**
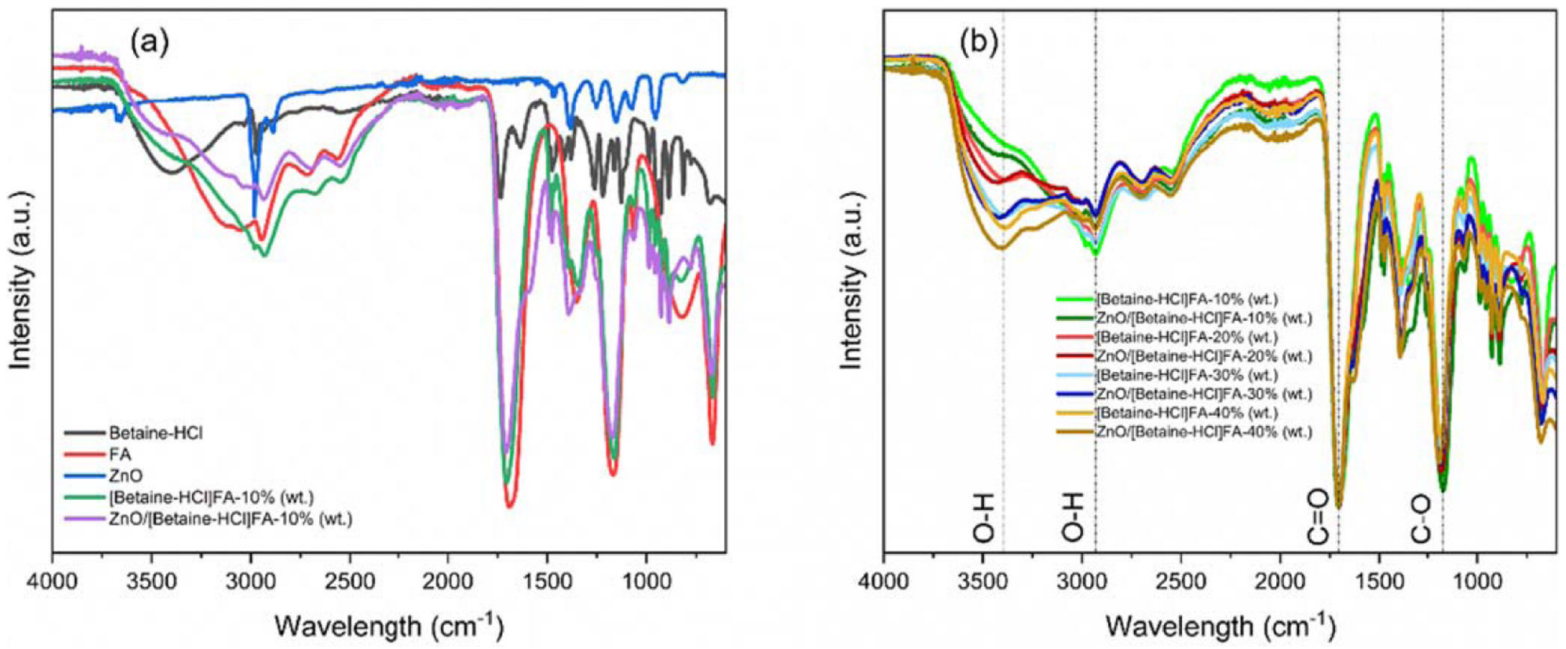
(a) FTIR spectra of ZnO in the DES ([Betaine-HCl][FA]) containing 10% water (b) Comparison of FTIR spectra of DES with different water concentrations in presence and absence of ZnO.

The dissolution mechanism of ZnO in DES was evaluated using FTIR and Raman spectroscopy. The shifts in the FTIR spectra during the formation of DES is shown in Fig. 1a. A blueshift in the –OH stretching at 2922 cm^−1^ in [Betaine-HCl]FA-10% water (DES_1_) from 2955 cm^−1^ (FA) is observed, which indicates the formation of hydrogen bond (–OH…Cl) between Cl^−^ of Betaine-HCl and –OH of FA.^17^ Furthermore, the C–O stretching peak (at 1158 cm^−1^) in FTIR spectrum shifted to lower wavenumber relative to the peak in FA (1165 cm^−1^) by 7 cm^−1^, and peaks at 1474 cm^−1^ and 954 cm^−1^ blueshifted from 1468 cm^−1^ and 944 cm^−1^ compared to Betaine-HCl, Fig. 1a. On addition of ZnO in DES_1_, further blueshift in C–O stretching occurs. The C=O stretching peak ~1694 cm^−1^ in FA shifts to a higher wavenumber in ZnO/DES_1_ (~1705 cm^−1^) possibly due to the disruption of hydrogen bond in the DES. Peak at 1633 cm^−1^ in Betaine-HCl which disappeared in the DES reappears in ZnO/DES_1_ as a shoulder at 1589 cm^−1^, indicating an interaction with ZnO. It has been postulated that during the dissolution of ZnO, O atom of formic acid may incorporate directly with Zn (II), while Cl^−^ ion from the HBA could participate in breaking the Zn–O bonds.^13^ Similarly, DES ([Betaine-HCl]FA) containing 20, 30, and 40% of water (DES_2_/DES_3_/DES_4_) showed blue shift of the –OH peak from 2955 to 2929 cm^−1^ which can be attributed to the formation of intermolecular hydrogen bonds, Fig. 1b.^20^ However, the intensity of the peak decreased with increase in the water content in the DES. Similar blueshift phenomenon was also observed by Ma et al.,^22^ with increase in water concentration in DES formed by mixing ChCl with tartaric acid (TA). In addition, the intensity of –OH at 3400 cm^−1^ increases with the increase in water content in DES, which indicates the modification of hydrogen bond in the DES with the addition of water.^20,22^ Furthermore, the intensity of C=O and C–O stretching peaks decrease with increase in water, which indicates that adding water in DES might have reduced the strength of hydrogen bonds.^8^

**Figure 2:**
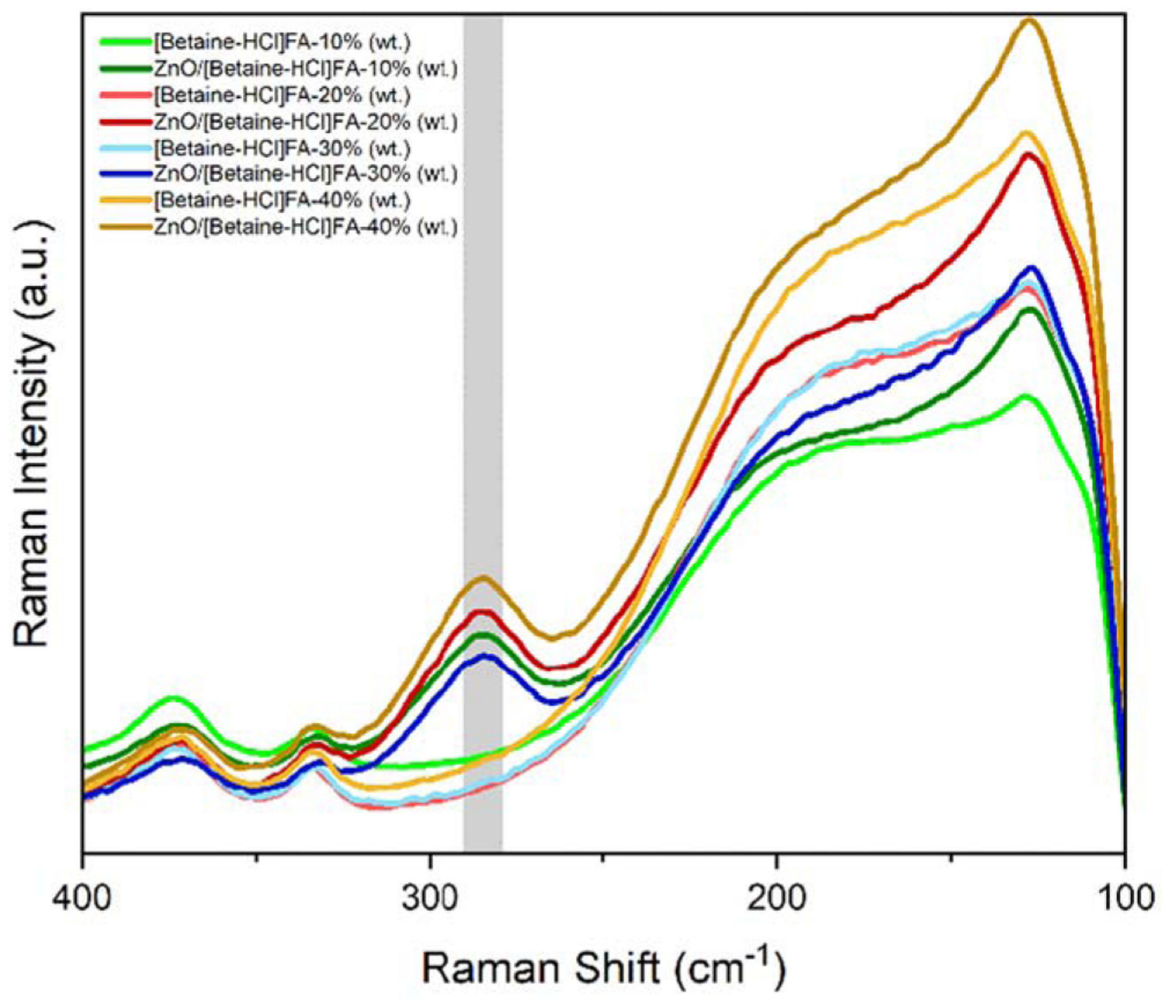
Comparison of Raman spectra of DES ([Betaine-HCl]FA) containing different concentrations of water and presence and absence of ZnO.

Raman spectroscopy was used to further understand the Zn-complex in DES. Comparing the Raman spectra in Fig. 2 of DES_1_ (light green curve), and ZnO/DES_1_ (dark green curve) between 400 and 100 cm^−1^, no significant change in the spectra is observed except the appearance of the new peak at 283 cm^−1^ for ZnO/DES_1_. Similarly, Raman spectra of ZnO/DES_2_, ZnO/DES_3_, ZnO/DES_4_ show an extra peak at 283 cm^−1^. The emergence of peak at 283 cm^−1^ can be attributed to the formation of Zn-complex through the coordination of Zn-Cl, which is in good agreement with previous studies.^6,8,23^ Therefore, it appears that Cl^−^ ion from the Betaine-HCl and formic acid may be the key factors to dissolve ZnO in the DES. The formation of zinc chloride complexes with different ratios of chloride to zinc has shown Raman bands at 277, 283, 300, and 385 cm^−1^ that was assigned to [ZnCl_4_]^2−^, [ZnCl_3_]^−^, [ZnCl_2_]^+^, [ZnCl]^+^, and [Zn(H_2_O)_6_]^2+^ respectively.^24^ Similarly, using ab initio calculations Parchment et al.^25^ showed the Raman band at 284 cm^−1^ corresponds to Zn-Cl stretching in [Zn(H_2_O)Cl_3_]^−^. Based on these studies and from the Raman spectra obtained in Betaine-HCl/FA/water system, the most possible zinc complex here could be [ZnCl_3_FA]^−^. Furthermore, it is evident that the addition of water does not shift the peak significantly, which confirms the stability of the Zn complex formed in the DES.

### Electrochemical analysis of DES and ZnO/DES

**Figure 3:**
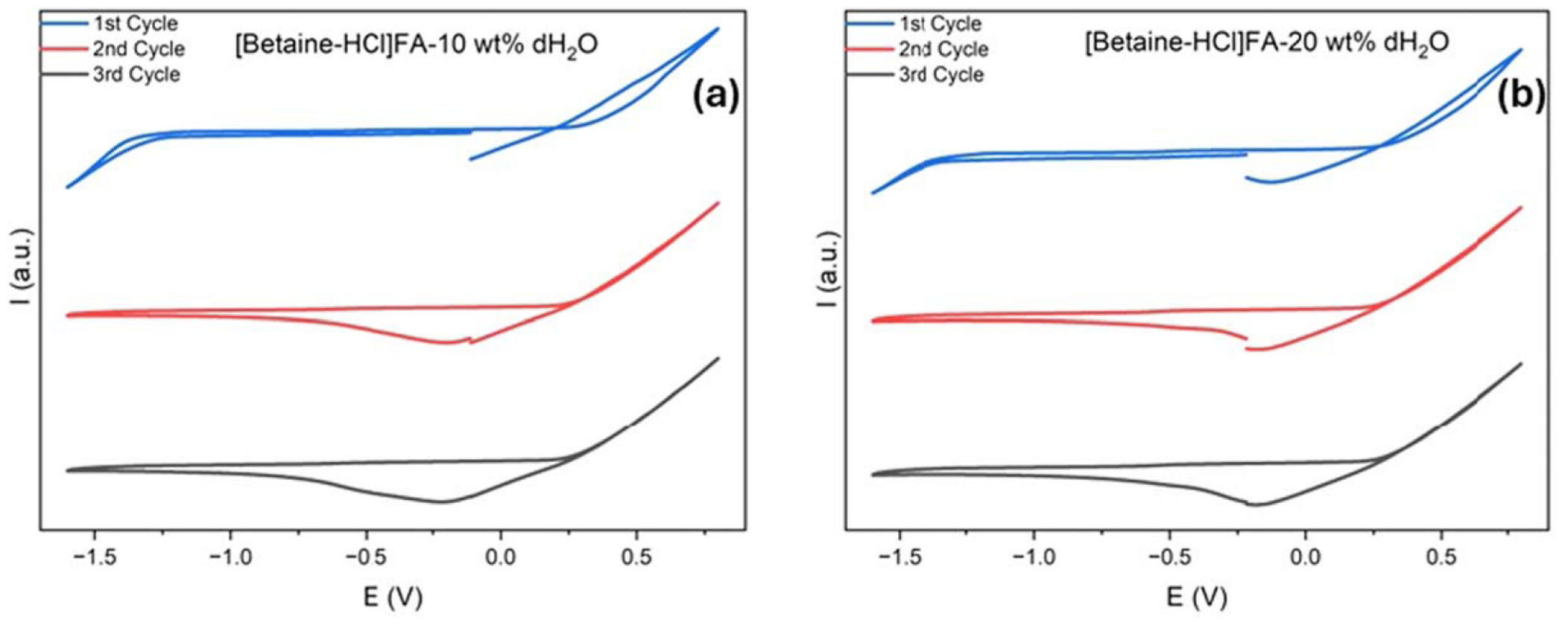
CV of [Betaine-HCl]FA containing (a) 10% water (b) 20% water using graphite as electrodes.

Cyclic voltammetry (CV) was used to assess the electrochemical behaviour of DES and ZnO/DES. CV of DES_1_ in the cathodic regime (Fig. 3a) shows an increase in negative current at −1.25 V which can be related to the decomposition of betaine in the first cycle. In the anodic scan, an increase in the current is observed from +0.25 V vs Pt quasi reference electrode (Fig. 3a).

**Figure 4:**
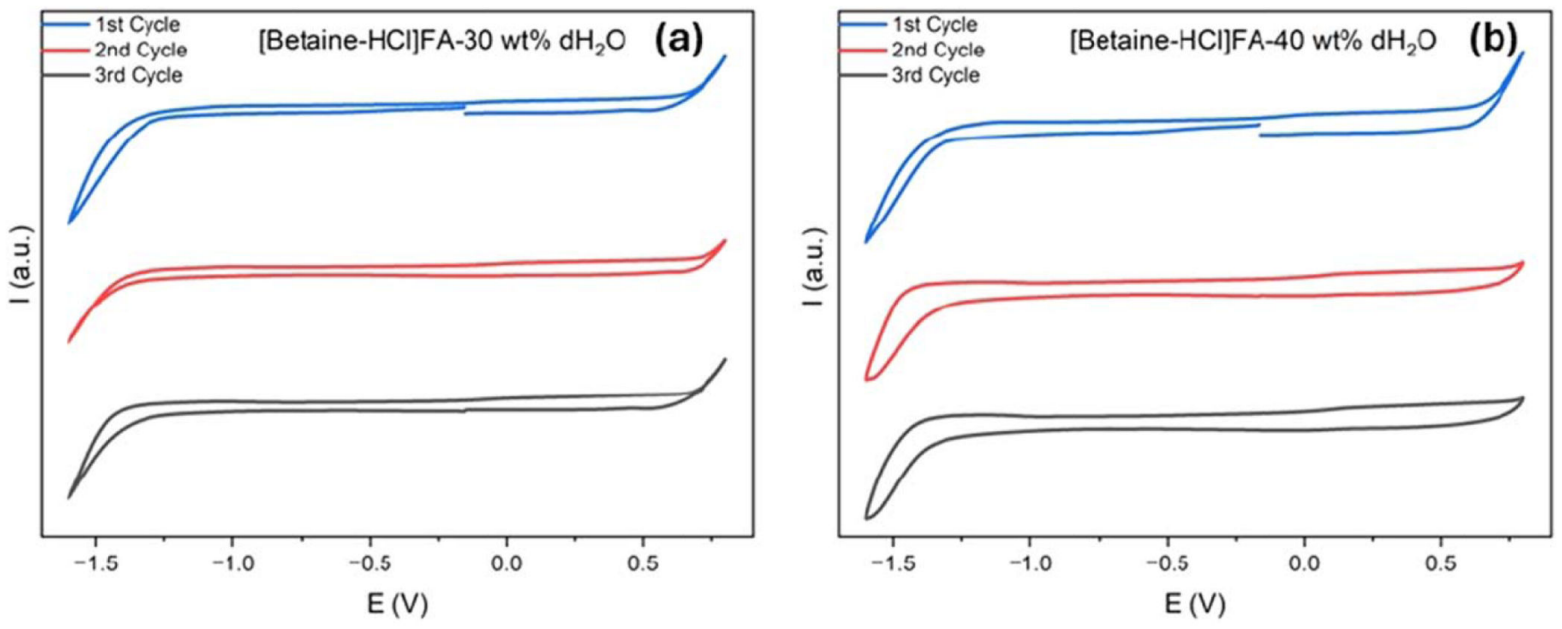
CV of [Betaine-HCl]FA containing (a) 30% water (b) 40% water using graphite as electrodes.

On reversing the scan at +0.75 V, a reduction peak at −0.2 V occurs which can be ascribed to the reduction of the oxidation products of the decomposed electrolyte. Interestingly, in the second cycle (red line, Fig. 3a) we do not see any decomposition of the DES even up to −1.5 V which indicates that passivation of the electrode has occurred in the first cycle. Similar behaviour of the CV is also seen in the third CV cycle. During the CV scan no bubbles on the working graphite electrode between +0.25 V to −1.25 V was observed. Based on the CV results, we can conclude that the potential window for DES_1_ is ~1.5 V. DES_2_ shows similar results as DES_1_ where the decomposition of the DES occurs at −1.25 V in the cathodic regime and +0.25 V in the anodic regime (Fig. 3b). Surprisingly, on increasing the water concentration to 30 and 40% (DES_3_ and DES_4_), an increase in the electrochemical window occurs (Figs. 4a and 4b).

It is evident from Figs. 4a and 4b, that the decomposition of the electrolyte shifts to −1.3 V in the cathodic regime and +0.6 V in the anodic regime. Furthermore, no reduction peak is observed within the electrochemical window due to the decomposition of the electrolyte. This leads to an increase in the electrochemical window in DES_3_ and DES_4_ to ~1.9 V.

**Figure 5:**
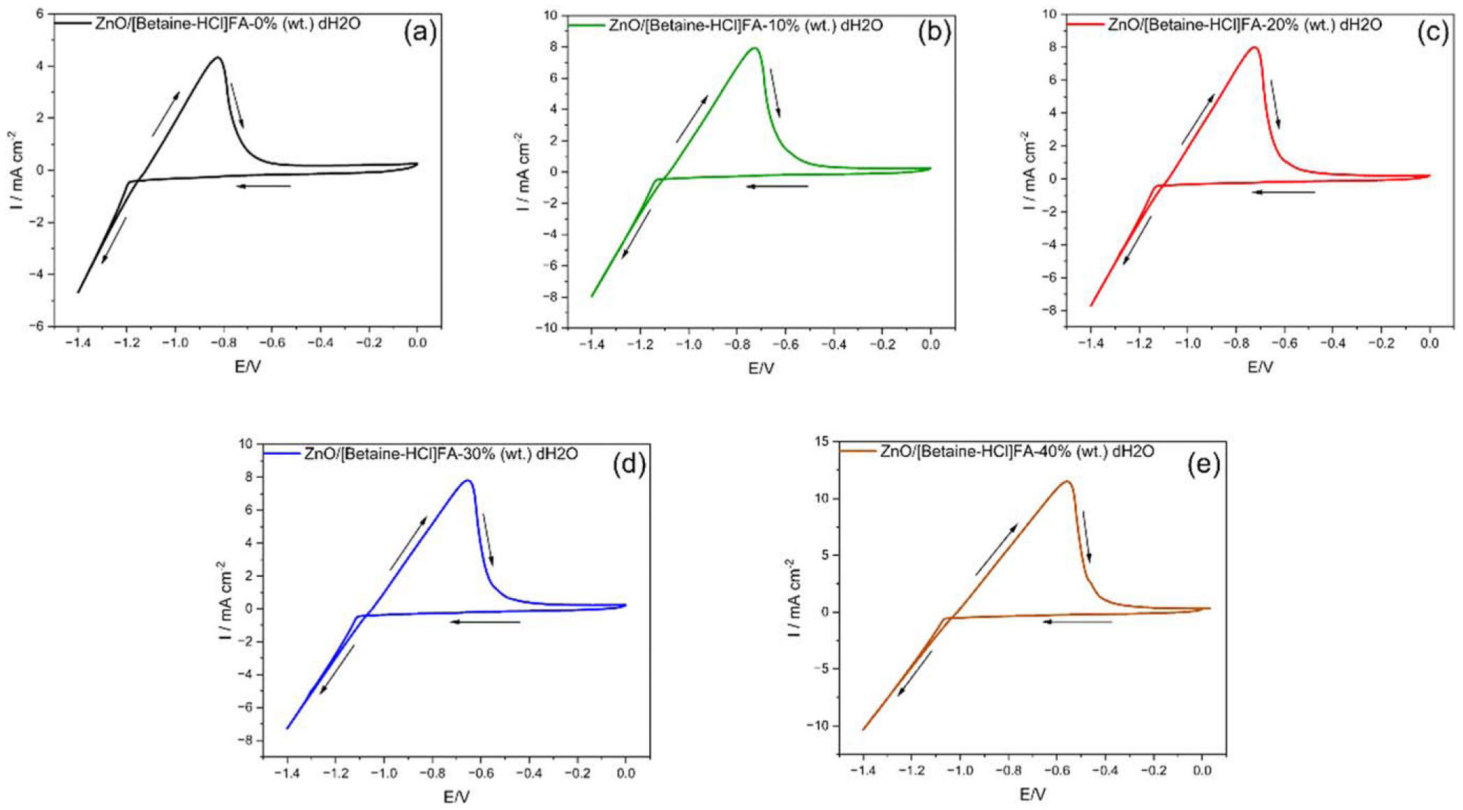
Cyclic voltammograms of (a) ZnO/[Betaine-HCl]FA (b) ZnO/[Betaine-HCl]FA-10 wt% dH_2_O (c) ZnO/[Betaine-HCl]FA-20 wt% dH_2_O (d) ZnO/[Betaine-HCl]FA-30 wt% dH_2_O (e) ZnO/[Betaine-HCl]FA-40 wt% dH_2_O containing saturated ZnO concentration at room temperature (294 K).

As it was found that the stability of DES improves on the addition of ZnO in the electrolyte, CV was performed initially on ZnO/DES. From Fig. 5a an increase in negative current is observed from −1.22 V which corresponds to the deposition of Zn. A corresponding oxidation peak at −0.75 V is observed which can be ascribed to the oxidation/stripping of the electrodeposited Zn.

These results are in good agreement with the previous studies in different DES electrolytes.^23,26,27^

On addition of water in the DES, the CV shows a slight shift in the reduction current to around −1.1 V (Fig. 5b) and an oxidation peak for Zn oxidation/stripping occurs around −0.7 V. Further addition of water in ZnO/DES shows a similar trend wherein the electrodeposition of Zn starts at −1.1 V (Figs. 5c–5e), whereas slight shift in the oxidation peak is noted. It is evident that the addition of water in DES clearly affects the current density as the negative current density is significantly higher compared to DES containing no water. Similarly, current density of stripping process in anodic regime increases with water. The presence of water in the DES decreases the viscosity of the electrolyte which leads to an increase in the diffusion of ions, thereby increasing the current density. Similar studies have been observed in other DES-water systems.^8,27^

### Characterisation of electrodeposits

The electrodeposition of Zn from the ZnO/DESs were performed at two different potentials (−1.2 and −1.4 V). The nucleation-growth process at different potentials could not be resolved based on the current-time transients and existing models (Fig. S2), possibly due to complex electrode/electrolyte interface in DES solvents.

**Figure 6:**
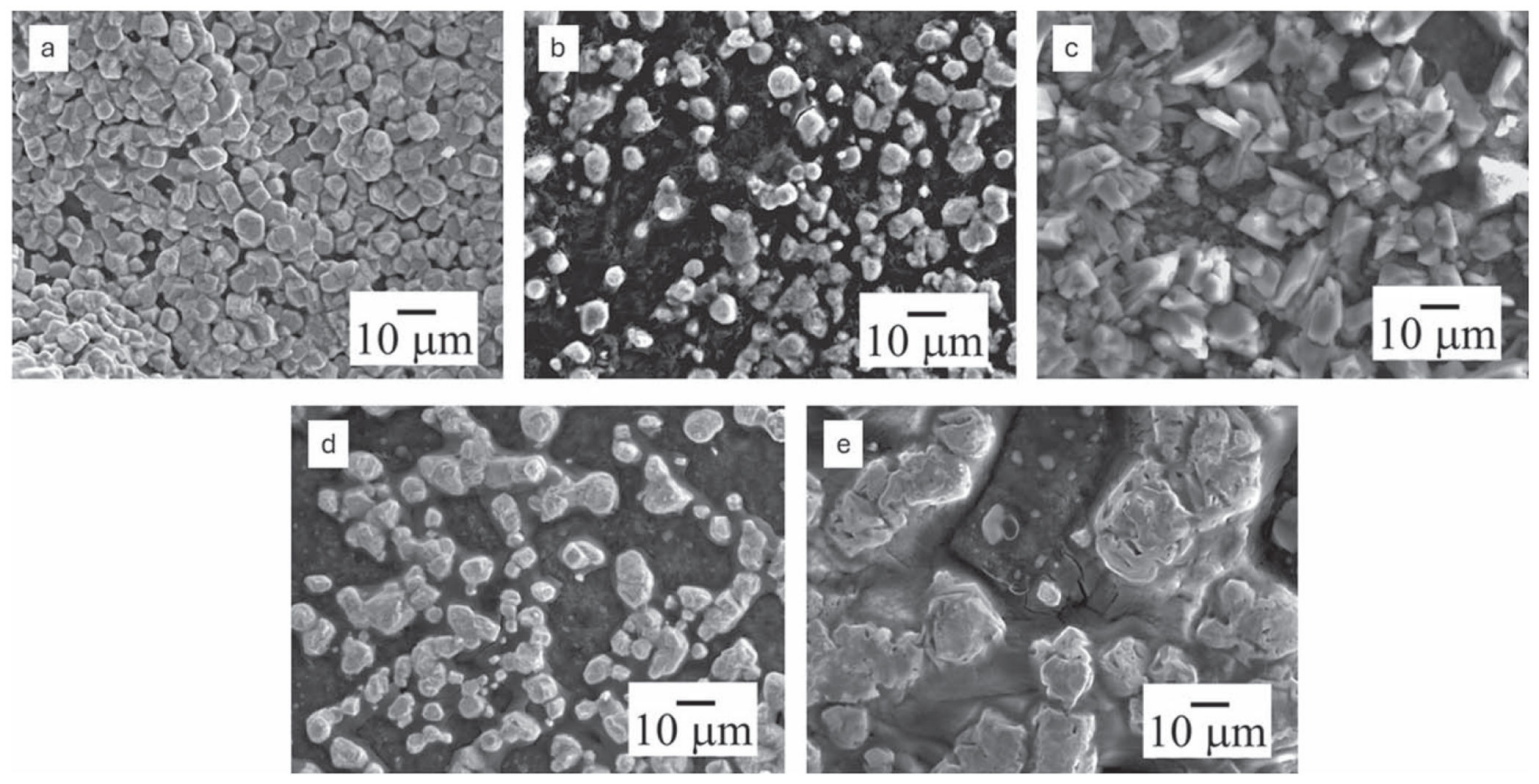
SEM images of Zn deposits obtained from (a) ZnO/[Betaine-HCl]FA (b) ZnO/[Betaine-HCl]FA-10 wt% dH_2_O (c) ZnO/[Betaine-HCl]FA-20 wt% dH_2_O (d) ZnO/[Betaine-HCl]FA-30 wt% dH_2_O (e) ZnO/[Betaine-HCl]FA-40 wt% dH_2_O at −1.2 V for 30 min at room temperature.

The effect of the applied potentials on the morphology of Zn electrodeposits was evaluated by SEM. The SEM of the Zn deposited at −1.2 V in the DES without water is shown in Fig. 6a. Uniform particles of 1–3 μm in size are observed in Fig. 6a. The addition of 10% water in the DES leads to non-uniform deposition of Zn particles in the size range of 3–5 μm (Fig. 6b). Interestingly, faceted particles in the size range of 4–6 μm are deposited from ZnO/DES_2_ at −1.2 V as seen in Fig. 6c. This indicates that an increase in the concentration of water in ZnO changes the nucleation/growth process. Non-uniform deposition occurs when Zn is deposited from ZnO/DES_3_ and ZnO/DES_3_ at −1.2 V (Figs. 6d and 6e) which can be attributed to the change in the electrode/electrolyte interfacial processes and Zn speciation affecting the Zn deposition process. Based on the current-time transition curves in Fig. S2, it is evident that DES containing 0, 10 and 20% water show similar I-t curves at −1.2 V whereas the I-t curves changes on addition of 30 and 40% water. This indicates that up to 20% water addition, the interface maybe governed by DES^8^ which leads to faceted Zn deposit whereas with higher water concentration, the interface is governed by aqueous interface which leads to non-inform deposits.

**Figure 7:**
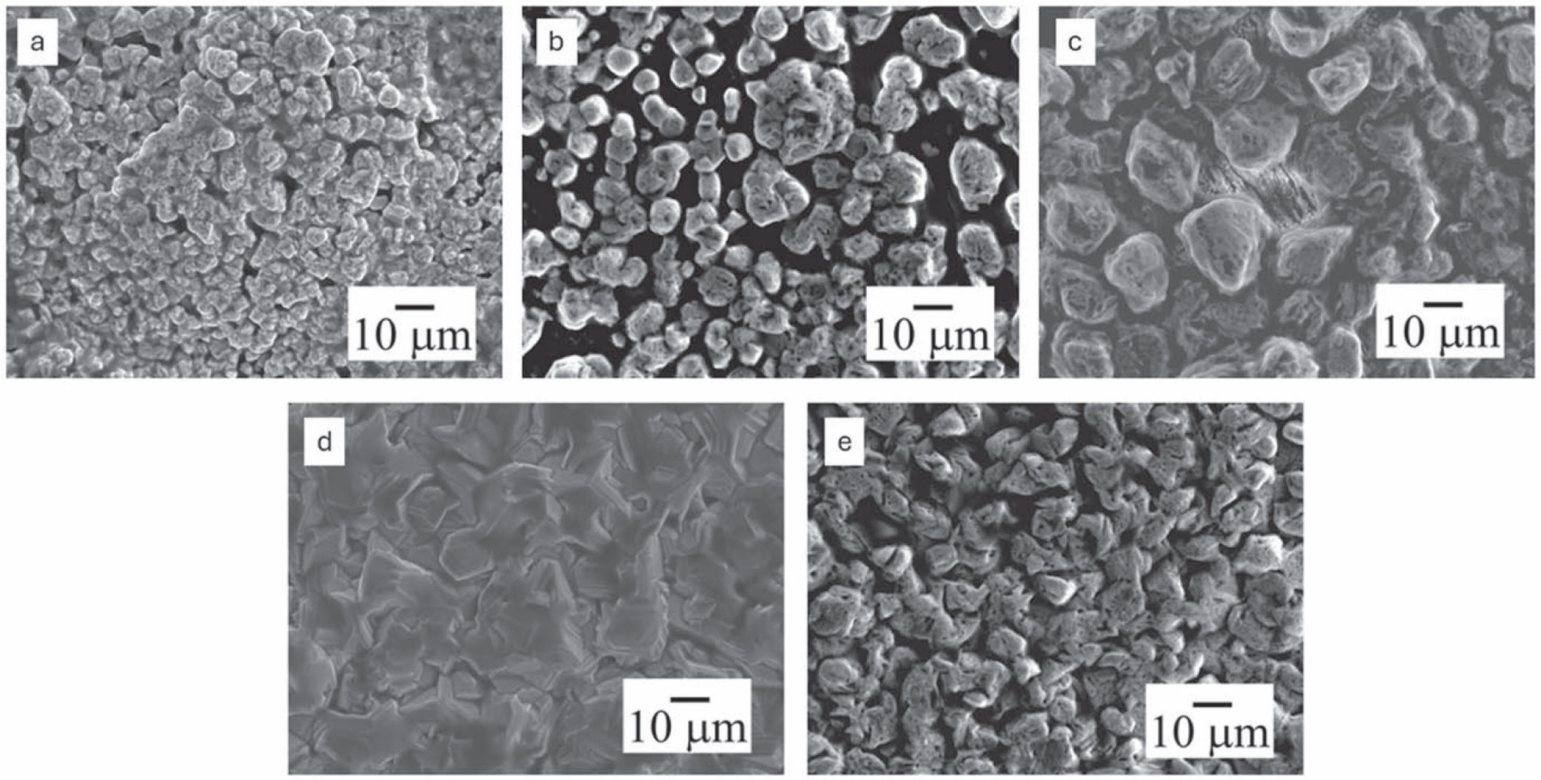
SEM images of Zn deposits obtained from (a) ZnO/[Betaine-HCl]FA (b) ZnO/[Betaine-HCl]FA-10 wt% dH_2_O (c) ZnO/[Betaine-HCl]FA-20 wt% dH_2_O (d) ZnO/[Betaine-HCl]FA-30 wt% dH_2_O (e) ZnO/[Betaine-HCl]FA-40 wt% dH_2_O at −1.4 V for 30 min at room temperature.

On electrodepositing at −1.4 V, the ZnO/DES without water shows agglomeration of Zn particles having 2–4 μm in size along with decomposed DES, Fig. 7a. Since applied potential is the main driving force for the electrodeposition of Zn, the change in the potential influences the nucleation-growth of particles which results in increase in the number of zinc nuclei and accelerates the growth of nuclei.^4,14^ On addition of water, Zn particles in the size range of 3–10 μm are observed at the same potential (Fig. 7b). On further increase in the water concentration, large Zn clusters in the size range of 10–20 μm are obtained from ZnO/DES_2_ which could be attributed to a change in the interfacial processes at the electrode/electrolyte interface (Fig. 7c). At higher water concentrations (ZnO/DES_3/4_) thin film deposits and faceted structures having particle size of 3–5 μm is obtained. Based on the studies, it is evident that both water concentration and electrode potential affect the Zn deposits possibly due to change in the interfacial processes and nucleation/growth processes of Zn on the graphite electrode.

**Figure 8:**
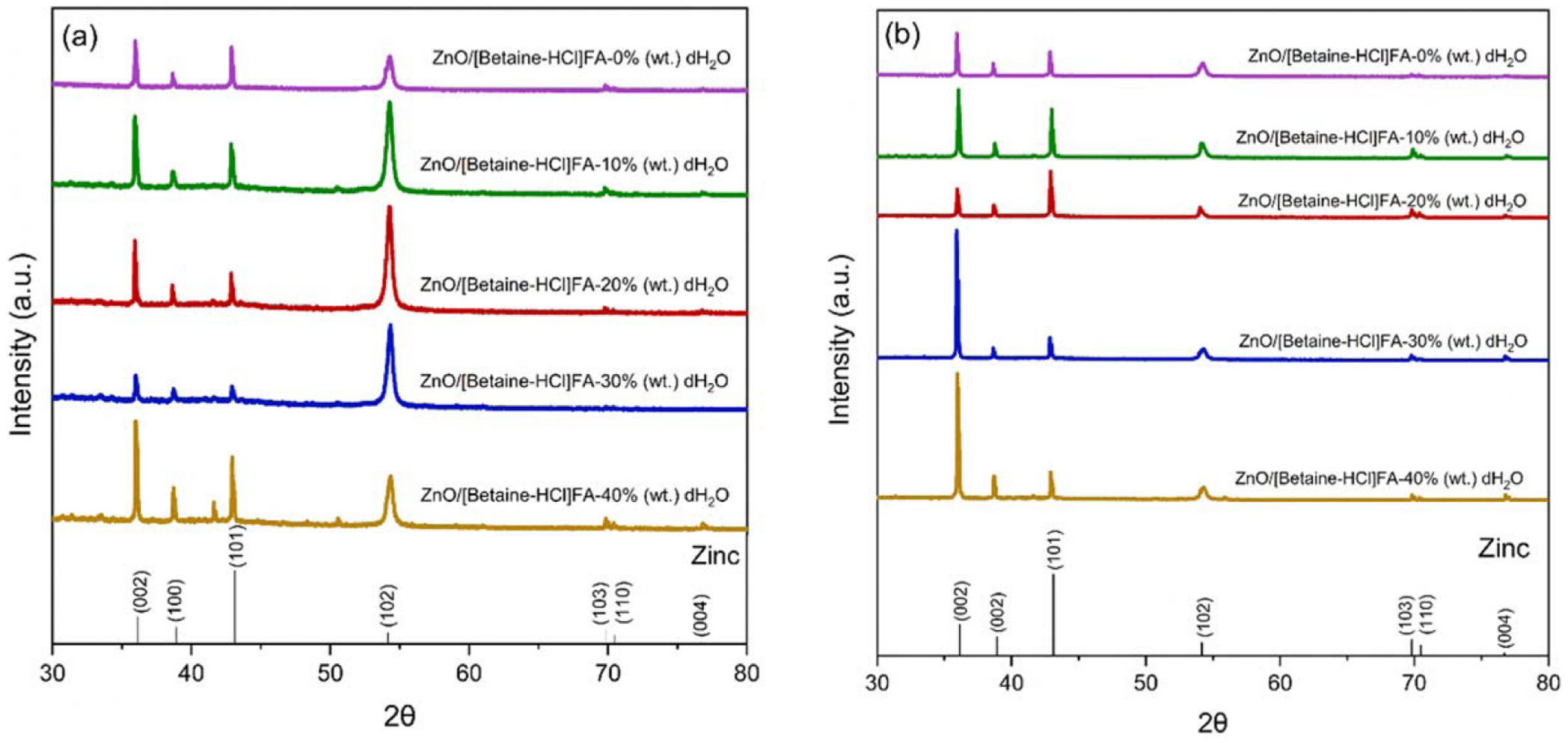
XRD images of Zn deposits obtained from (a) ZnO/[Betaine-HCl]FA containing different water concentrations at −1.2 V and (b) at −1.4 V for 30 min at room temperature.

XRD of the electrodeposits obtained at −1.2 V from DES containing different concentrations of water are compared in Fig. 8a which represents characteristic peak of Zn (ICDD 78–9363). Comparing the Zn electrodeposits at −1.2 V, it is evident that the growth plane of Zn electrodeposits is affected by the presence of water. The peak intensity ratio of 101 to 002 plane (Table ST1 in supporting information) varies with the concentration of water, which indicates that the nucleation/growth process during the electrodeposition has changed.^28^ It has been shown that Zn (100) and Zn (101) show high self-diffusion barrier whereas Zn (002) show high chemical stability as their surface energy is lower than that of (100) and (101) planes.^28^

The XRD of the electrodeposits obtained at −1.4 V from ZnO in [Betaine-HCl]FA (DES) containing different concentrations of water is shown in Fig. 8b, which is similar to that obtained at −1.2 V. Comparing the diffraction pattern, it is evident that at higher water concentrations (30 and 40%), Zn primarily grows along 002 plane which indicates that an increase in water concentration improves the chemical stability of Zn on depositing at more negative potentials.

## Conclusions

Here, we show an environmentally benign ([Betaine-HCl][FA]) electrolyte which can dissolve >1 mole ZnO depending on the water concentration. FTIR spectra identified the formation of hydrogen bond during the formation of DES and the dissolution of ZnO in DES, whereas Raman spectra indicated the possible formation of [ZnCl_3_FA]^−^ after dissolving ZnO into DES. Interestingly, cyclic voltammetry showed that electrochemical window of the DES increases with increase in water concentration. Electrodeposition from the DES electrolytes showed that Zn morphology depended on the electrode potential and the water concentration. Uniform deposits and Zn thin films was obtained on depositing at more negative potentials. Thus, this process shows a sustainable route to recycle Zn from various ZnO sources including spent zinc-air battery.

## Supplementary Material

Supplementary data

## Figures and Tables

**Table I:** Characteristics of formic acid-based deep eutectic solvents (DES).

Formic acid-based DES	Molar ratio of betaine chloride (HBA) to formic acid (HBD)	Water content (%, w/w)	Viscosity (mPa. s)	Solubility (g l^−1^)
[Betaine-HCl]FA-10% dH_2_O (DES_1_)	1:6	10	6.059 ± 0.056	85
[Betaine-HCl]FA-20% dH_2_O (DES_2_)	1:6	20	3.148 ± 0.020	75
[Betaine-HCl]FA-30% dH_2_O (DES_3_)	1:6	30	2.829 ± 0.001	71
[Betaine-HCl]FA-40% dH_2_O (DES_4_)	1:6	40	2.399 ± 0.001	70

## Data Availability

The link to the data is provided in 10.17633/rd.brunel.28376195.v1.
