# Supplementary material for: Electrochemical and Spectroscopic Studies of Zinc Oxide in an Eco-Friendly Deep Eutectic Solvent for Zn Electrodeposition
Source: J Electrochem Soc. Author manuscript; Available in PMC 2025 Aug 19. (PMC7618030; doi:10.1149/1945-7111/adda79)
Supplement: Supplementary data [file EMS207655-supplement-Supplementary_data.docx]

Supporting Information

**Electrochemical and spectroscopic studies of Zinc oxide in an eco-friendly Deep Eutectic Solvent for Zn electrodeposition**

Kazem Mohammadzadeh^1^, Uttam Kumer Roy^1,2^, Abhishek Lahiri^1^

^1^Department of Chemical Engineering, College of Engineering, Design and Physical Science, Brunel University London, Kingston Lane, Uxbridge, Middlesex, UB8 3PH, UK

^2^Water Engineering and Development Centre, School of Architecture, Building and Civil Engineering, Loughborough University, Epinal Way, Loughborough, Leicestershire, LE11


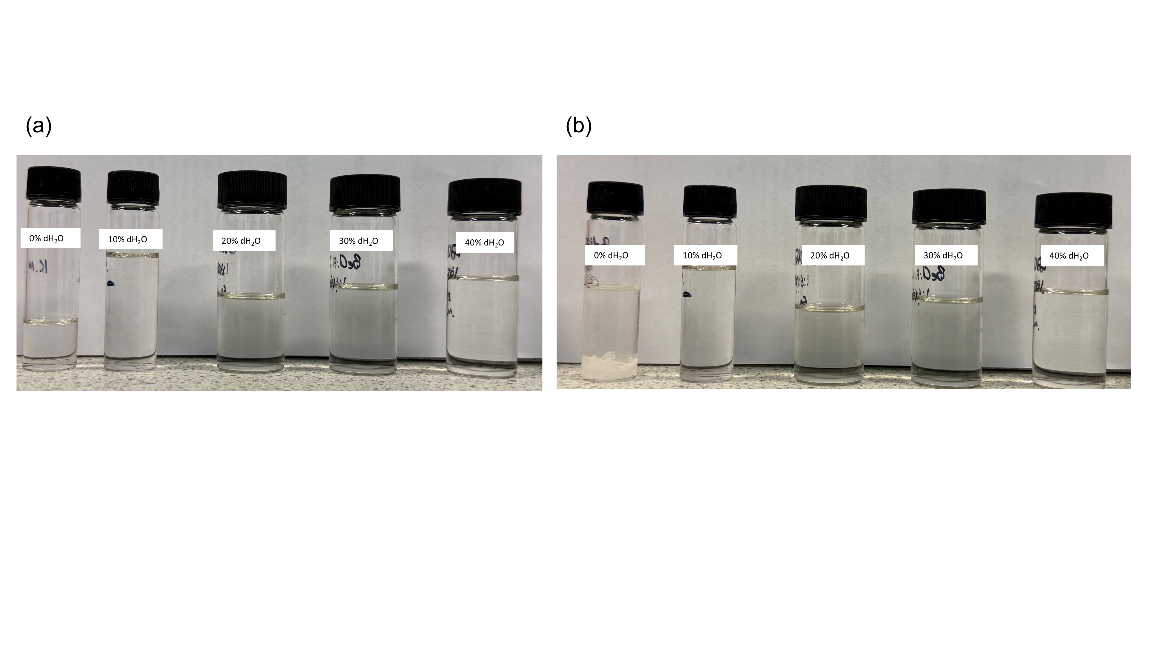


Figure S1: (a) Freshly prepared DES with different water concentrations (b) DES after one day


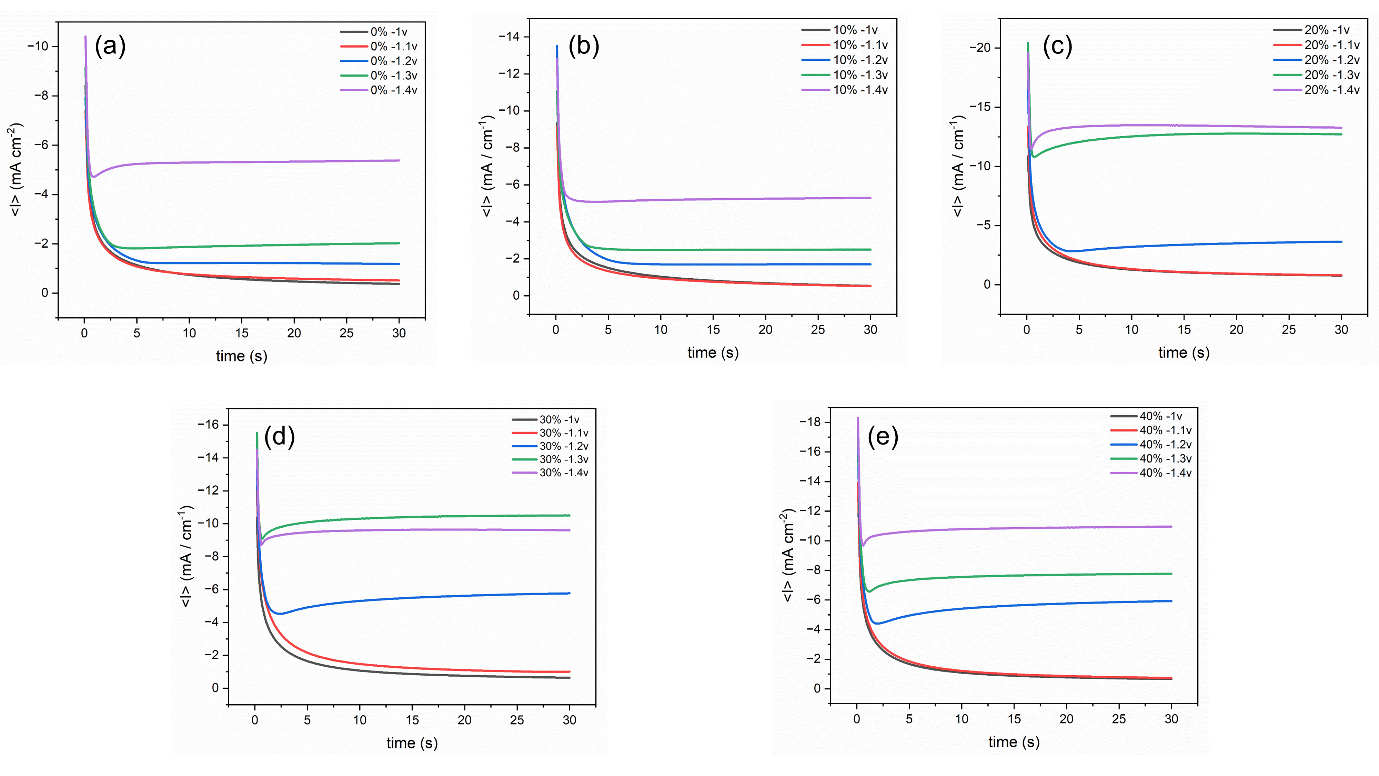


Figure S2: Chronoamperometric current–time (i–t) transients recorded in five DES electrolytes at different potentials containing (a) 0, (b) 10 (c) 20 (d) 30 and (e) 40 wt% water.

Table ST1 relative intensity ratio of the major planes for deposits at -1.2v and -1.4v.

|  | Plane 101/ Plane 002 for deposition at -1.2v | Plane 101/ Plane 002 for deposition at -1.4v |
| --- | --- | --- |
| DES-0% (wt.) dH2O | 0.88181 | 0.58507 |
| DES-10% (wt.) dH2O | 0.66767 | 0.71808 |
| DES-20% (wt.) dH2O | 0.58167 | 1.62512 |
| DES-30% (wt.) dH2O | 0.72229 | 0.18223 |
| DES-40% (wt.) dH2O | 0.68147 | 0.22876 |
